# The Impact of Disability on the Lives of Children; Cross-Sectional Data Including 8,900 Children with Disabilities and 898,834 Children without Disabilities across 30 Countries

**DOI:** 10.1371/journal.pone.0107300

**Published:** 2014-09-09

**Authors:** Hannah Kuper, Adrienne Monteath-van Dok, Kevin Wing, Lisa Danquah, Jenny Evans, Maria Zuurmond, Jacqueline Gallinetti

**Affiliations:** 1 International Centre for Evidence in Disability, Department of Clinical Research, London School of Hygiene & Tropical Medicine, London, United Kingdom; 2 Plan International, Woking, Surrey, United Kingdom; 3 Non Communicable Disease Epidemiology Department, London School of Hygiene & Tropical Medicine, London, United Kingdom; University of Perugia, Italy

## Abstract

**Background:**

Children with disabilities are widely believed to be less likely to attend school or access health care, and more vulnerable to poverty. There is currently little large-scale or internationally comparable evidence to support these claims. The aim of this study was to investigate the impact of disability on the lives of children sponsored by Plan International across 30 countries.

**Methods and Findings:**

We conducted a cross-sectional survey including 907,734 children aged 0–17 participating in the Plan International Sponsorship Programme across 30 countries in 2012. Parents/guardians were interviewed using standardised questionnaires including information on: age, sex, health, education, poverty, and water and sanitation facilities. Disability was assessed through a single question and information was collected on type of impairment. The dataset included 8,900 children with reported disabilities across 30 countries. The prevalence of disability ranged from 0.4%–3.0% and was higher in boys than girls in 22 of the 30 countries assessed – generally in the range of 1.3–1.4 fold higher. Children with disabilities were much less likely to attend formal education in comparison to children without disabilities in each of the 30 countries, with age-sex adjusted odds ratios exceeding 10 for nearly half of the countries. This relationship varied by impairment type. Among those attending school, children with disabilities were at a lower level of schooling for their age compared to children without disabilities. Children with disabilities were more likely to report experiencing a serious illness in the last 12 months, except in Niger. There was no clear relationship between disability and poverty.

**Conclusions:**

Children with disabilities are at risk of not fulfilling their educational potential and are more vulnerable to serious illness. This exclusion is likely to have a long-term deleterious impact on their lives unless services are adapted to promote their inclusion.

## Introduction

The World Report on Disability – published in 2011 by the World Bank and WHO - estimates that there are more than one billion people globally living with disabilities [1]. This includes approximately 93 million children aged 0–14 years living with “moderate or severe disability” (5·1%) of whom 13 million (0·7%) experience severe difficulties. Others have put this figure even higher – with UNICEF estimating that there were 150 million children with disabilities globally in 2005 [2]. Both groups agree that childhood disability is most common in low and middle income countries [1], [2]. However, there are few underlying data supporting these figures. The 2013 UNICEF State of the World’s Children Report which focussed on childhood disability noted the “global estimates are essentially speculative”, and we are still reliant on out-dated estimates [3].

The few data that exist show that children with disabilities face barriers to participation in many activities [1]. Children with disabilities are less likely to start school, have lower rates of school attendance and lower transition rates to higher levels of education. The gap in school attendance associated with disability observed at the primary level widens further at the secondary level [4]. Furthermore, the overall quality of the educational experiences of disabled children is often inadequate where they do attend school [5]. Children with disabilities may also have poorer access to health services [6], while experiencing higher health care needs [7]–[9]. Overall, there is a perceived lack of inclusion children with disabilities in the development agenda [3].

These exclusions are contrary to the spirit of two key conventions relevant to children with disabilities: the UN Convention on the Rights of the Child [10], and the UN Convention on the Rights of Persons with Disabilities [11]. Countries that have ratified these conventions, which include almost all countries in the world, are responsible for including children with disabilities in key areas (e.g. education). Reliable data are needed on the prevalence and impact of childhood disability in order to develop these inclusive services. There have been efforts to collect these data, but these studies are often too small to make robust inferences, measure disability inconsistently, and do not assess participation comprehensively. One example of a large and internationally comparable study is within the context of the UNICEF Multiple Indicator Cluster Survey which screened children across 18 countries [8], [12]. While providing valuable data on disability, these surveys included only children 2–9 years and did not comprehensively assess the implications for service delivery. There is therefore an urgent need for more large-scale studies about childhood disability.

Plan International is one of the oldest and largest children’s rights and development organisations in the world, working in 50 low and middle income countries. Plan collects data annually on the more than 1·4 million children in their sponsorship programme, including on disability, and therefore provides an excellent opportunity to fill some of our knowledge gaps about childhood disability. The aim of this study was to investigate the impact of disability on the lives of children sponsored by Plan International across 30 countries.

## Methods

### Ethical approval

Oral consent is sought from the parent/guardian and signed for by the interviewer. Written consent is not sought as many of the participants are illiterate. Oral consent is documented by the interviewer who signs the statement “I confirm that I discussed these issues with the family and they willingly agreed to them”. Ethical approval for the study was granted by the London School of Hygiene & Tropical Medicine. Data are anonymised, and do not include names or location data. This consent procedure was approved by the ethics committees.

### Study participants

Participants in the Plan International Sponsorship Programme include children aged 0–18 across 49 countries. Historically there have been varied reasons for children entering into Plan’s sponsorship programme, but in recent years this is based on criteria for poverty and development. Only one child is sponsored per family and the sponsored child must live with his/her parent(s) or guardian. The sponsorship programme focuses on a specific region or district in each country and the areas of work are defined locally.

### Data collection

The sponsorship interview takes place approximately once per year (varying from twice per year to once in 18 months), so that the information can be used to update the sponsor on the child’s status. The sponsorship data are collected in the local language through Plan interviewers using paper questionnaires. The interview takes place with the caretaker of the sponsored child, usually the mother.

The same questionnaire is used in each country. It covers the following areas: age, sex, birth registration, health, education, type of house and assets, and water and sanitation facilities. Since 2011 the caregiver is asked “Does the sponsored child have an impairment/a medical condition that can lead to disability?”. If the answer is “yes” then the respondent is asked about the type and duration of impairment.

The interviewers are trained in data collection methods and provided with standard guidelines. The training consists of explaining consent and the questionnaire and practical training. The interviewers are supervised by local Plan sponsorship managers.

### Data entry

Data are entered into a purpose-built database. Each child is assigned a unique sponsorship number, which ensures the anonymity of the child and his/her family. The database holds data starting from 2008, however for the current analyses only the sponsored children interviewed in 2012 data were included since these include the disability assessment.

### Data analysis

Data analyses were restricted to the 30 countries that included at least 100 children with self-reported disabilities within the sponsorship programme. Descriptive analyses were undertaken to estimate and describe the age and sex of the cohort, the proportion of children with a reported disability, and the proportion of each type of disability. Principal component analysis was used to compute a poverty score for each country, based upon economic proxy variables (family assets and housing characteristics) [13].

The following variables were analysed for an association with disability status for each country: sex, school attendance, school level, serious illness in the last 12 months, water and sanitation, and poverty score. Univariable analysis was performed by cross-tabulating each variable against disability status and calculating unadjusted odds ratios of association. Age was considered an *a-priori* confounder of any observed associations, and stratification by sex was performed to investigate whether there were clear differences in the relationship between disability and each variable for boys compared with girls. A simple multivariable analysis was then conducted for each country, comparing children with disabilities to those without with respect to each variable, while adjusting for age (continuous variable) and sex where appropriate. Analyses were restricted to children aged 0–17, given the small number of young people aged 18 years and above in the sponsorship programme. The analysis for the education variables were restricted to children aged five years and above.

All analyses were performed using STATA (version 13·1).

## Results

The analyses include 907,734 children aged 0–17 across 30 countries (Table 1). The number of children per country ranged from 6,443 in Rwanda to 65,360 in India. The average age was usually between 9 and 10 years. Girls made up the largest proportion of participants within each country.

**Table 1:** Socio-demographic characteristics of children in the Plan Sponsorship programme.

Country	Number of children	Average age (SD^1^)	% Girls
South America			
Bolivia	41,979	9·5 (4·2)	59%
Brazil	12,993	6·9 (4·0)	56%
Colombia	22,020	8·8 (4·3)	58%
Dominican Republic	26,560	8·7 (4·7)	59%
Ecuador	47,070	9·7 (4·2)	54%
El Salvador	34,814	10·1 (4·0)	55%
Guatemala	38,797	9·9 (3·9)	56%
Honduras	34,040	9·3 (4·2)	56%
Nicaragua	27,793	9·5 (4·3)	54%
Paraguay	7,813	9·0 (4·1)	54%
Peru	25,364	8·9 (4·5)	58%
Africa			
Benin	24,547	10·5 (3·2)	94%
Egypt	33,871	9·9 (4·0)	56%
Guinea	28,208	9·9 (3·9)	70%
Kenya	60,139	10·1 (3·9)	58%
Mozambique	6,782	6·2 (2·3)	56%
Niger	19,103	7·7 (4·3)	67%
Rwanda	6,443	7·2 (3·3)	71%
Senegal	32,738	9·1 (4·2)	64%
Sudan	27,225	9·8 (4·0)	63%
Tanzania	24,303	9·8 (3·9)	59%
Uganda	35,466	9·6 (4·1)	61%
Zambia	16,725	10·2 (3·8)	53%
Zimbabwe	33,346	10·5 (3·9)	66%
Asia			
India	65,360	8·0 (4·1)	65%
Indonesia	45,860	9·4 (4·0)	55%
Nepal	38,450	9·6 (3·9)	74%
Philippines	33,543	9·6 (4·3)	61%
Sri Lanka	21,743	9·6 (4·3)	55%
Vietnam	34,639	8·5 (3·9)	66%

1 SD: standard deviation.

The dataset included 8,900 children with self-reported disabilities across the 30 countries (Table 2). The prevalence of disability was 0·98% (0·96–1·00%) overall, ranging from a low of 0·4% in Benin, Kenya and Tanzania, to a high of 3·3% in Rwanda. There was evidence that the prevalence of disability was higher in boys than girls in 22 of the 30 countries assessed – generally in the range of 1·3–1·4 fold higher. In the remaining 8 countries prevalence was similar between boys and girls. The dominant types of impairment were physical, vision and communication impairment (Table S1). Hearing impairment was relatively rare, while learning impairment was more common in Latin American countries than in African or Asian countries.

**Table 2:** Prevalence of disabilities among Plan’s sponsored children, by country and sex.

Region/country	No. of childrenwith disabilities	Prevalence(95% CI^1^)	Prevalence males(95% CI)	Prevalence females(95% CI)	Age-adjusted OR comparing malesto females (95% CI^2^)
South America					
Bolivia	372	0·9% (0·8–1·0%)	1·0% (0·9–1·2%)	0·8% (0·7–0·9%)	1·2 (1·0–1·5)
Brazil	143	1·1% (0·9–1·3%)	1·4% (1·1–1·7%)	0·8% (0·6–1·1%)	1·6 (1·2–2·2)
Colombia	235	1·0% (0·9–1·2%)	1·3% (1·0–1·6%)	0·9% (0·7–1·0%)	1·4 (1·1–1·9)
Dominican Rep	178	0·7% (0·6–0·8%)	0·8% (0·6–0·9%)	0·6% (0·5–0·7%)	1·2 (0·9–1·6)
Ecuador	793	1·7% (1·6–1·8%)	1·9% (1·8–2·1%)	1·7% (1·6–1·8%)	1·3 (1·1–1·5)
El Salvador	646	1·9% (1·7–2·0%)	2·2% (2·0–2·4%)	1·6% (1·4–1·7%)	1·4 (1·2–1·6)
Guatemala	432	1·1% (1·0–1·2%)	1·3% (1·1–1·5%)	1·1% (0·8–1·1%)	1·3 (1·1–1·6)
Honduras	551	1·6% (1·5–1·8%)	2·0% (1·8–2·2%)	1·3% (1·1–1·5%)	1·5 (1·2–1·7)
Nicaragua	459	1·7% (1·5–1·8%)	1·9% (1·7–2·1%)	1·4% (1·2–1·6%)	1·3 (1·1–1·6)
Paraguay	114	1·5% (1·2–1·7%)	1·9% (1·4–2·3%)	1·1% (0·7–1·4%)	1·7 (1·1–2·4)
Peru	195	0·8% (0·7–0·9%)	0·9% (0·7–1·1%)	0·7% (0·5–0·8%)	1·3 (1·0–1·8)
Africa					
Benin	108	0·4% (0·4–0·5%)	0·3% (0·04–0·6%)	0·4% (0·4–0·5%)	1·0 (0·9–1·0)
Egypt	452	1·3% (1·2–1·5%)	1·6% (1·4–1·8%)	1·2% (1·0–1·3%)	1·4 (1·1–1·6)
Guinea	146	0·5% (0·4–0·6%)	0·5% (0·4–0·7%)	0·5% (0·4–0·6%)	1·3 (0·9–1·8)
Kenya	258	0·4% (0·4–0·5%)	0·5% (0·4–0·6%)	0·4% (0·3–0·4%)	1·3 (1·0–1·6)
Mozambique	119	1·8% (1·4–2·1%)	1·8% (1·3–2·3%)	1·7% (1·3–2·1%)	1·1 (0·7–1·5)
Niger	185	1·0% (0·8–1·1%)	1·4% (1·1–1·7%)	0·8% (0·6–0·9%)	1·6 (1·2–2·1)
Rwanda	214	3·3% (2·8–3·8%)	3·8% (2·9–4·7%)	3·1% (2·6–3·6%)	1·2 (0·9–1·6)
Senegal	155	0·5% (0·4–0·5%)	0·7% (0·6–0·9%)	0·3% (0·3–0·4%)	1·9 (1·3–2·6)
Sudan	131	0·5% (0·4–0·6%)	0·7% (0·5–0·8%)	0·4% (0·3–0·5%)	1·6 (1·1–2·2)
Tanzania	105	0·4% (0·3–0·4%)	0·5% (0·4–0·6%)	0·4% (0·3–0·5%)	1·2 (0·8–1·7)
Uganda	268	0·8% (0·7–0·8%)	0·8% (0·7–1·0%)	0·7% (0·6–0·8%)	1·2 (0·9–1·5)
Zambia	113	0·7% (0·6–0·8%)	0·8% (0·6–0·9%)	0·6% (0·4–0·8%)	1·4 (1·0–1·8)
Zimbabwe	200	0·6% (0·5–0·7%)	0·7% (0·6–0·9%)	0·5% (0·4–0·6%)	1·4 (1·0–1·8)
Asia					
India	522	0·8% (0·7–0·9%)	1·0% (0·9–1·1%)	0·7% (0·6–0·8%)	1·3 (1·1–1·5)
Indonesia	376	0·8% (0·7–0·9%)	1·0% (0·8–1·1%)	0·7% (0·6–0·8%)	1·4 (1·1–1·7)
Nepal	259	0·7% (0·6–0·8%)	1·0% (0·8–1·2%)	0·5% (0·5–0·6%)	1·9 (1·5–2·4)
Philippines	397	1·2% (1·1–1·3%)	1·4% (1·2–1·6%)	1·0% (0·9–1·2%)	1·3 (1·1–1·6)
Sri Lanka	166	0·8% (0·6–0·9%)	0·8% (0·6–0·9%)	0·8% (0·6–0·9%)	1·0 (0·7–1·3)
Vietnam	608	1·8% (1·6–2·0%)	2·5% (2·2–2·8%)	1·3% (1·2–1·5%)	1·7 (1·5–2·0)

1 CI: Confidence interval.

2 OR: Odds ratio.

Age adjusted analysis showed that there were no substantial differences in the associations when comparing boys and girls within each country and so the unstratified multivariable results were presented.

Almost all children aged 5 and above without disabilities sponsored by Plan were attending formal education. In contrast, among children with disabilities generally 30–40% were not attending school, but this ranged from 17% in Zimbabwe to 78% in Guinea. As a result, there was strong evidence from the age-adjusted analyses that children with disabilities were much less likely to attend formal education in comparison to children without disabilities in each of the 30 countries (Table 3). For seven countries the OR was below 5, for nine countries it was 5–10, for eight it was 11–20 and for six the OR was over 20. In the majority of countries, the most frequent reason for not attending school was reported as “having an impairment” among children with disabilities, and being “too young” for the children without disabilities (Table S2).

**Table 3:** Effect of disability on school attendance amongst Plan’s sponsored children aged 5 years and above.

Country	Attend formaleducation	Children withdisabilities	Children withoutdisabilities	Age and sex adjustedOR^1^ (95% CI^2^)
South America				
Bolivia	Yes	228 (66%)	33718 (94%)	Baseline
	No	120 (34%)	2204 (6%)	8·1 (6·5–10·2)
Brazil	Yes	85 (75%)	8717 (98%)	Baseline
	No	29 (25%)	181 (2%)	19·5 (12·3–31·0)
Colombia	Yes	149 (70%)	17679 (98%)	Baseline
	No	63 (30%)	316 (2%)	26·1 (18·6–36·7)
Dominican Rep	Yes	93 (60%)	19437 (95%)	Baseline
	No	63 (40%)	1050 (5%)	22·6 (15·8–32·4)
Ecuador	Yes	231 (64%)	26995 (95%)	Baseline
	No	128 (36%)	1546 (5%)	12·9 (10·0–16·7)
El Salvador	Yes	402 (66%)	27941 (91%)	Baseline
	No	210 (34%)	2611 (9%)	5·7 (4·8–6·7)
Guatemala	Yes	242 (59%)	30264 (87%)	Baseline
	No	167 (41%)	4696 (13%)	4·6 (3·8–5·6)
Honduras	Yes	319 (62%)	24228 (85%)	Baseline
	No	192 (38%)	4435 (15%)	3·6 (2·9–4·3)
Nicaragua	Yes	243 (57%)	21791 (92%)	Baseline
	No	181 (43%)	1838 (8%)	9·6 (7·8–11·8)
Paraguay	Yes	56 (54%)	6333 (96%)	Baseline
	No	47 (46%)	250 (4%)	21·5 (14·3–32·5)
Peru	Yes	116 (67%)	19595 (96%)	Baseline
	No	57 (33%)	728 (4%)	14·8 (10·6–20·8)
Africa				
Benin	Yes	84 (79%)	21838 (91%)	Baseline
	No	23 (21%)	2100 (9%)	3·3 (2·1–5·3)
Egypt	Yes	146 (36%)	27330 (92%)	Baseline
	No	258 (64%)	2268 (8%)	22·2 (18·0–27·3)
Guinea	Yes	32 (22%)	18193 (71%)	Baseline
	No	111 (78%)	7445 (29%)	9·2 (6·2–13·7)
Kenya	Yes	177 (74%)	53393 (99%)	Baseline
	No	61 (26%)	454 (1%)	56·5 (40·8–78·2)
Mozambique	Yes	75 (74%)	4190 (84%)	Baseline
	No	26 (26%)	770 (16%)	3·6 (2·1–5·9)
Niger	Yes	57 (33%)	9391 (68%)	Baseline
	No	116 (67%)	4482 (32%)	4·1 (2·9–5·6)
Rwanda	Yes	128 (74%)	4040 (86%)	Baseline
	No	44 (26%)	659 (14%)	2·7 (1·8–4·1)
Senegal	Yes	41 (28%)	17552 (64%)	Baseline
	No	108 (72%)	9976 (36%)	5·1 (3·5–7·3)
Sudan	Yes	70 (55%)	22240 (91%)	Baseline
	No	58 (45%)	2094 (9%)	17·1 (11·8–24·7)
Tanzania	Yes	76 (74%)	20349 (92%)	Baseline
	No	27 (26%)	1668 (8%)	4·7 (3·0–7·4)
Uganda	Yes	159 (66%)	29281 (96%)	Baseline
	No	81 (34%)	1374 (4%)	13·1 (9·8–17·5)
Zambia	Yes	69 (62%)	13855 (90%)	Baseline
	No	43 (38%)	1562 (10%)	6·7 (4·6–9·9)
Zimbabwe	Yes	158 (83%)	29456 (96%)	Baseline
	No	32 (17%)	1125 (4%)	5·4 (3·7–8·0)
Asia				
India	Yes	316 (68%)	47035 (92%)	Baseline
	No	149 (32%)	4013 (8%)	5·6 (4·5–6·9)
Indonesia	Yes	192 (55%)	37888 (95%)	Baseline
	No	157 (45%)	2149 (5%)	14·0 (11·3–17·4)
Nepal	Yes	169 (74%)	32265 (95%)	Baseline
	No	60 (26%)	1720 (5%)	8·2 (6·0–11·2)
Philippines	Yes	231 (64%)	26995 (95%)	Baseline
	No	128 (36%)	1546 (5%)	12·9 (10·0–16·7)
Sri Lanka	Yes	101 (67%)	18132 (98%)	Baseline
	No	49 (33%)	319 (2%)	36·3 (24·2–54·4)
Vietnam	Yes	406 (74%)	27274 (97%)	Baseline
	No	146 (26%)	850 (3%)	13·8 (10·9–17·5)

1 OR: Odds ratio.

2 CI: Confidence interval.

Stratifying the relationship between disability and school attendance by impairment type revealed important patterns (Table 4). In comparison to children without disabilities, children with learning or communication impairments were consistently among the least likely to attend school, particularly in Africa. In many countries children with physical impairments were similarly excluded from education, while in other countries this pattern was less apparent. Children with vision or hearing impairment were generally the most likely to attend school among children with disabilities.

**Table 4:** Age and sex-adjusted odds ratio (95% Confidence Interval) for the association between disability and formal education attendance among Plan’s sponsored children aged 5 years and above: comparator children without disabilities.

Country	Learning OR^1^(95% CI^2^)	Physical OR^1^(95% CI^2^)	Communication OR^1^(95% CI^2^)	Vision OR^1^(95% CI^2^)	Hearing OR^1^(95% CI^2^)
South America					
Bolivia	11·6 (7·3–18·6)	4·8 (2·7–8·7)	15·6 (10·7–22·8)	2·5 (1·3–5·0)	5·7 (2·5–12·8)
Brazil	20·3 (8·6–48·0)	29·2 (14·1–60·5)	32·1 (11·5–90·0)	–	28·6 (4·9–165·8)
Colombia	32·6 (18·0–59·1)	30·0 (15·1–59·7)	69·7 (37·2–130·4)	5·9 (1·7–20·3)	–
Dominican Rep	51·3 (19·8–132·5)	104·7 (51·0–215·3)	185·1 (74·5–460·1)	2·7 (0·9–8·2)	18·8 (3·2–111·8)
Ecuador	32·5 (15·0–70·3)	13·9 (9·0–21·7)	29·5 (18·0–48·3)	2·8 (1·2–6·6)	11·2 (3·6–34·8)
El Salvador	13·9 (8·3–23·4)	4·8 (3·6–6·4)	9·1 (6·9–12·1)	2·2 (1·2–3·8)	1·6 (0·6–4·0)
Guatemala	18·3 (9·4–35·6)	5·8 (3·8–8·7)	6·6 (4·6–9·5)	1·3 (0·8–2·2)	2·4 (1·0–5·6)
Honduras	5·0 (3·5–7·3)	8·3 (5·3–13·0)	7·6 (4·9–11·8)	0·9 (0·6–1·4)	2·4 (1·1–5·1)
Nicaragua	12·4 (8·0–19·1)	23·3 (14·9–36·6)	15·5 (10·5–22·9)	2·5 (1·5–4·3)	1·5 (0·4–5·1)
Paraguay	38·2 (16·1–90·7)	34·6 (16·5–72·3)	40·5 (17·8–91·8)	1·3 (0·2–10·0)	6·7 (0·7–60·6)
Peru	27·1 (14·1–51·9)	17·5 (8·4–36·7)	20·6 (10·9–39·2)	6·1 (2·5–15·2)	–
Africa					
Benin	–	1·8 (0·7–4·6)	23·0 (3·4–157·2)	2·5 (1·1–5·5)	11·7 (4·1–33·1)
Egypt	39·5 (25·0–62·3)	23·1 (16·1–33·3)	44·3 (27·8–70·7)	2·7 (1·3–5·6)	7·7 (2·8–21·3)
Guinea	–	5·2 (3·1–8·8)	39·7 (12·3–128·2)	4·7 (1·8–12·1)	28·8 (3·7–227·3)
Kenya	59·8 (14·9–240·2)	132·3 (70·1–249·7)	142·3 (78·1–259·5)	9·4 (2·7–32·3)	61·9 (30·8–124·3)
Mozambique	–	3·6 (1·2–10·4)	14·9 (5·7–38·6)	4·7 (1·0–23·3)	0·8 (0·2–3·7)
Niger	–	3·2 (2·0–5·1)	7·8 (3·8–15·7)	1·3 (0·6–3·2)	11·1 (3·2–39·0)
Rwanda	26·8 (3·0–236·6)	4·2 (2·2–8·1)	13·4 (3·6–49·1)	0·8 (0·3–2·2)	2·9 (1·1–8·0)
Senegal	–	3·2 (1·7–6·1)	11·1 (5·6–21·9)	1·8 (0·8–4·0)	6·5 (1·2–34·0)
Sudan	48·6 (9·3–253·5)	68·0 (34·9–132·3)	54·0 (24·1–120·9)	2·5 (0·7–9·3)	36·8 (9·5–141·9)
Tanzania	47·9 (4·1–559·5)	3·6 (1·9–6·9)	7·5 (2·9–19·7)	2·4 (0·5–11·5)	8·8 (2·6–30·5)
Uganda	56·3 (5·1–616·6)	17·6 (10·8–28·8)	35·8 (20·9–61·4)	3·7 (1·2–11·5)	5·0 (2·4–10·5)
Zambia	33·2 (6·2–176·2)	6·3 (3·1–12·9)	20·5 (9·0–46·9)	3·4 (1·3–9·0)	3·7 (1·4–10·3)
Zimbabwe	25·1 (10·2–61·8)	3·9 (1·8–8·2)	10·3 (5·2–20·4)	0·9 (0·1–6·9)	0·9 (0·1–6·6)
Asia					
India	16·5 (6·2–44·1)	5·4 (3·9–7·5)	10·9 (7·2–16·5)	2·4 (1·4–4·0)	2·7 (0·8–8·8)
Indonesia	4·3 (1·9–10·0)	13·0 (8·8–19·2)	30·8 (21·8–43·6)	4·5 (2·2–9·4)	4·6 (1·9–11·4)
Nepal	–	6·0 (3·8–9·6)	28·0 (15·9–49·4)	3·6 (1·4–9·7)	–
Philippines	32·5 (15·0–70·3)	13·9 (9·0–21·7)	29·5 (18·0–48·3)	2·8 (1·2–6·6)	11·2 (3·6–34·8)
Sri Lanka	62·2 (18·5–209·0)	66·1 (28·5–153·3)	189·9 (86·0–419·3)	6·2 (1·9–19·9)	8·4 (2·2–32·9)
Vietnam	26·0 (16·0–42·2)	10·8 (7·0–16·8)	61·9 (36·3–105·4)	3·8 (2·0–7·5)	10·6 (4·8–23·6)

1 OR: Age and sex-adjusted odds ratio in comparison to children without disabilities.

2 CI: Confidence interval.

Among children attending school, those with disabilities were more likely to be at nursery or primary level, rather than secondary or above, in comparison to children without disabilities (Table 5). This meant that the children with disabilities were at a lower level of schooling for their age compared to children without disabilities. This pattern was generally less apparent within African countries, as opposed to countries from Latin America or Asia.

**Table 5:** Age and sex-adjusted odds ratio (95% Confidence Interval) for the association between disability and school level among Plan’s sponsored children aged 5 years and above attending formal education: Comparator children in secondary education or above.

Country	Primary education (compared to secondary+ education)	Nursery education (compared to secondary+ education)
South America		
Bolivia	2·4 (1·5–3·7)	5·0 (2·3–10·7)
Brazil	6·3 (2·5–15·4)	10·4 (3·1–35·2)
Colombia	8·1 (4·5–14·7)	29·6 (11·0–79·7)
Dominican Rep	2·2 (0·9–5·0)	2·1 (0·5–8·4)
Ecuador	3·0 (1·9–4·6)	5·1 (2·3–11·4)
El Salvador	2·3 (1·7–3·2)	3·1 (1·7–5·6)
Guatemala	2·4 (1·5–4·0)	6·1 (2·9–13·0)
Honduras	2·6 (1·7–3·8)	4·6 (2·4–8·8)
Nicaragua	5·2 (3·4–7·9)	14·9 (7·2–30·8)
Paraguay	3·1 (0·7–13·8)	2·6 (0·3–19·3)
Peru	3·0 (1·5–5·7)	5·5 (2·0–15·2)
Africa		
Benin	4·7 (1·4–15·6)	–
Egypt	2·0 (1·2–3·3)	1·9 (0·5–6·5)
Guinea	2·1 (0·3–16·6)	1·6 (0·1–31·6)
Kenya	3·2 (0·4–23·0)	3·3 (0·4–27·0)
Mozambique	0·6 (0·2–2·0)	–
Niger	0·5 (0·2–1·3)	0·6 (0·1–3·2)
Rwanda	0·4 (0·2–1·1)	0·3 (0·1–1·1)
Senegal	1·3 (0·5–3·5)	2·7 (0·4–19·1)
Sudan	1·9 (0·8–4·6)	2·4 (0·4–14·2)
Tanzania	2·4 (0·8–7·0)	2·0 (0·5–8·6)
Uganda	3·9 (1·2–12·7)	4·3 (1·1–17·3)
Zambia	1·6 (0·6–4·1)	1·2 (0·2–6·0)
Zimbabwe	2·1 (1·2–3·5)	2·8 (1·0–7·8)
Asia		
India	2·0 (1·4–2·9)	3·0 (1·7–5·5)
Indonesia	2·9 (1·8–4·7)	3·5 (1·5–8·5)
Nepal	2·6 (1·5–4·3)	5·6 (2·4–12·7)
Philippines	3·0 (1·9–4·6)	5·1 (2·3–11·4)
Sri Lanka	1·7 (1·0–2·8)	3·6 (1·3–9·5)
Vietnam	2·4 (1·7–3·5)	3·5 (1·9–6·4)

There was evidence that children with disabilities were more likely to report experiencing a serious illness in the last 12 months in comparison to children without disabilities, in 29 of the countries (not Niger) (Table 6). For 16 countries, the OR was below 5, for 10 countries it was between 5 and 10, and for 3 countries the OR was above 10. The types of illness included those that were impairment related (e.g. eye problems) as well as general illnesses (e.g. malaria, acute respiratory infection). Malnutrition was relatively rare, yet there was good evidence for an association with disability in Brazil, Colombia, Ecuador, Guatemala, Honduras, India, Indonesia, and Vietnam. The vast majority of children (generally >97%) sought treatment when ill, and so it was not possible to assess the relationship between treatment uptake and disability for most countries. However, disability was associated with not seeking care when ill in Rwanda, Senegal and Zambia.

**Table 6:** Association between disability and serious illness in the last 12 months among Plan’s sponsored children.

Country	Serious illness	Children with disabilities	Children without disabilities	Age and sex adjusted OR^1^ (95% CI^2^)
South America				
Bolivia	No	270 (73%)	38583 (93%)	Baseline
	Yes	102 (27%)	3035 (7%)	5·0 (4·0–6·3)
Brazil	No	129 (90%)	12559 (98%)	Baseline
	Yes	15 (10%)	291 (2%)	5·3 (3·0–9·1)
Colombia	No	204 (87%)	21397 (98%)	Baseline
	Yes	31 (13%)	388 (2%)	8·6 (5·8–12·7)
Dominican Rep	No	163 (92%)	26051 (99%)	Baseline
	Yes	15 (8%)	331 (1%)	7·8 (4·5–13·4)
Ecuador	No	339 (85%)	31618 (95%)	Baseline
	Yes	58 (15%)	1528 (5%)	3·7 (2·8–5·0)
El Salvador	No	523 (81%)	31391 (92%)	Baseline
	Yes	123 (19%)	2777 (8%)	2·8 (2·3–3·4)
Guatemala	No	365 (84%)	36717 (96%)	Baseline
	Yes	67 (16%)	1648 (4%)	4·3 (3·3–5·6)
Honduras	No	398 (72%)	29342 (88%)	Baseline
	Yes	153 (28%)	4147 (12%)	2·9 (2·4–3·5)
Nicaragua	No	339 (74%)	23638 (86%)	Baseline
	Yes	121 (26%)	3699 (14%)	2·4 (1·9–3·0)
Paraguay	No	87 (76%)	7092 (92%)	Baseline
	Yes	27 (24%)	607 (8%)	3·8 (2·5–6·0)
Peru	No	162 (83%)	24189 (96%)	Baseline
	Yes	33 (17%)	983 (4%)	5·3 (3·6–7·7)
Africa				
Benin	No	89 (82%)	23401 (96%)	Baseline
	Yes	19 (18%)	1044 (4%)	4·7 (2·8–7·7)
Egypt	No	433 (96%)	33296 (100%)	Baseline
	Yes	19 (4%)	123 (0%)	12·1 (7·4–19·8)
Guinea	No	99 (68%)	27134 (97%)	Baseline
	Yes	47 (32%)	928 (3%)	14·3 (10·0–20·4)
Kenya	No	183 (71%)	54285 (91%)	Baseline
	Yes	75 (29%)	5597 (9%)	4·0 (3·1–5·3)
Mozambique	No	55 (46%)	4806 (72%)	Baseline
	Yes	64 (54%)	1857 (28%)	3·2 (2·3–4·7)
Niger	No	109 (59%)	11921 (63%)	Baseline
	Yes	76 (41%)	6998 (37%)	1·2 (0·9–1·6)
Rwanda	No	67 (31%)	4724 (76%)	Baseline
	Yes	147 (69%)	1505 (24%)	8·2 (6·1–11·2)
Senegal	No	126 (81%)	31733 (97%)	Baseline
	Yes	29 (19%)	855 (3%)	8·5 (5·6–12·8)
Sudan	No	113 (86%)	26362 (97%)	Baseline
	Yes	18 (14%)	734 (3%)	6·7 (4·0–11·2)
Tanzania	No	90 (86%)	23537 (97%)	Baseline
	Yes	15 (14%)	670 (3%)	5·7 (3·3–9·9)
Uganda	No	166 (62%)	28706 (82%)	Baseline
	Yes	102 (38%)	6492 (18%)	3·5 (2·6–4·6)
Zambia	No	67 (59%)	12367 (74%)	Baseline
	Yes	46 (41%)	4255 (26%)	2·2 (1·5–3·2)
Zimbabwe	No	155 (78%)	31759 (96%)	Baseline
	Yes	45 (23%)	1390 (4%)	6·6 (4·7–9·3)
Asia				
India	No	495 (95%)	63461 (98%)	Baseline
	Yes	27 (5%)	1377 (2%)	3·1 (2·1–4·7)
Indonesia	No	310 (82%)	43028 (95%)	Baseline
	Yes	66 (18%)	2458 (5%)	3·7 (2·9–4·9)
Nepal	No	227 (88%)	37090 (97%)	Baseline
	Yes	32 (12%)	1103 (3%)	4·7 (3·2–6·9)
Philippines	No	339 (85%)	31618 (95%)	Baseline
	Yes	58 (15%)	1528 (5%)	3·7 (2·8–5·0)
Sri Lanka	No	133 (80%)	21292 (99%)	Baseline
	Yes	33 (20%)	285 (1%)	19·3 (13·0–28·9)
Vietnam	No	460 (76%)	30367 (89%)	Baseline
	Yes	148 (24%)	3664 (11%)	3·2 (2·6–3·8)

1 OR: odds ratio.

2 CI: Confidence interval.

For most of the countries there was no association between being in the poorest quartile of poverty and disability (Table S3). For some countries there was evidence of a positive association (e.g. Paraguay, Peru, Egypt) but for the majority this relationship was inverse indicating a protective effect of poverty on disability (e.g. Ecuador, Benin, Uganda, India, Philippines).

## Discussion

This large data analysis conducted across 30 countries found that children with disabilities included within Plan International’s sponsorship programme were far less likely to attend school than children without disabilities. When they did attend school their level of schooling was below that of their same aged peers. The exclusion from schooling varied by impairment type so that children with learning or communication impairments were least likely to attend school, while those with hearing or visual impairments generally fared better. Children with disabilities were also much more likely to report having a serious illness in the last 12 months. In terms of socio-demographic differences, boys were more likely to be classed as having a disability, but there was no clear relationship between disability and poverty in this population.

The exclusion of children with disabilities from schooling reported in our study is consistent with findings from others across the globe [4], [8], [14], [15], including the World Health Surveys [1]. While the World Health Surveys reported that children with physical impairment generally fared better than those with intellectual or sensory impairments, we did not observe this pattern [1]. Exclusion from education has an immediate impact on a child in terms of exclusion from social participation, reduced personal well-being and welfare, and likely dependence on a family member for care during school hours [16]. The long-term impact may be even more profound. In Bangladesh, the cost of foregone income from lack of schooling and employment of people with disabilities and their caregivers is estimated at US$1·2 billion annually, or 1·7% of Gross Domestic Product [17]. The impact may also span across generations, as a study in Vietnam showed that children are less likely to go to school if they have a parent with a disability [15].

These analyses showed a strong relationship between disability and serious illness. Intriguingly, a link was demonstrated between disability and malnutrition in some countries, although the numbers were small. Other studies have suggested that children with disabilities are vulnerable to malnutrition [7], [18], although a large study by UNICEF including nearly 200,000 children across 15 studies showed that disability was linked to nutritional deficiency in eight of the countries, but not in the remainder [8]. The vast majority of children attended for treatment when ill, and so there were insufficient numbers to assess the impact of disability on access to health care. Other studies have demonstrated the existence of barriers to uptake of health and rehabilitation services by children with disabilities in low income settings [6], [19], [20]. This discrepancy may have arisen because illness was defined as “serious” in this study, while other studies may have included any illnesses.

A link between poverty and childhood disability could arise as a result of the direct costs (e.g. health/rehabilitation costs) or indirect costs (e.g. foregone parental earnings). We did not demonstrate a relationship between disability and poverty in this study. A large review found that the relationship between childhood disability and socio-economic circumstances was “inconsistent and inconclusive” across 24 primary studies from low and middle income countries [21]. Others report that poverty remains a major problem in safeguarding the wellbeing of children with disabilities: with up to 88% of caregivers unable to meet the basic needs of their children with disabilities [21]. The link between poverty and disability may not have been apparent in this study because the children were all in a sponsorship programme, and therefore were all poor, or because the sponsorship programme itself may have alleviated the impact of disability on poverty.

The higher prevalence of disability in boys as compared to girls was a consistent finding across the countries. This finding tallies with the higher child mortality rate observed among boys in most parts of the world (excepting India and China) [22], and the higher proportion of boys identified with disabilities in the UNICEF survey [8].

Support for the key findings of the study is given by the important methodological strengths in the design. The analyses were conducted in a very large data set, which included internationally comparable data across 30 countries. Multiple domains of inclusion were assessed with respect to impact of disability, such as education, health, and poverty. We therefore believe that this study makes an original and high quality scientific contribution, particularly in contrast to many of the previously conducted studies on childhood disability which are often small in scale, do not assess impact as broadly, and do not allow international comparisons to be made.

In terms of limitations, Plan’s sponsorship programmes are located in economically disadvantaged areas and sponsored children and their families are amongst the poorest or most marginalised within their communities. Consequently, the children in the analyses are not representative of the general population in the country and so it is not possible to make general inferences. This should not, however, compromise the internal validity of the findings. In addition, the prevalence of disability was relatively low among sponsored children in comparison to general estimates from the World Report on Disability [1], potentially because of the relatively restrictive way disability was measured leading to under-reporting as the parent/guardian may not identify the child as having a disability. Only one type of impairment could be reported per child and there was no clinical validation of self-report. There were missing data for key variables so that these could not be assessed, including birth registration, vaccination coverage and duration of disability. The variation in prevalence and type of disability by country implies that the interpretation of the disability question or the selection of children with disabilities into the programme varied, and we therefore did not believe that it was appropriate to conduct multi-level analyses. The impact of childhood disability often extends into adulthood as well as to other household members, and this was not assessed in the current study. However, the sponsorship database can be used in the future to track the life course of children with disabilities longitudinally and further household level research could fill these research gaps.

A central implication of our findings is for the need for renewed focus on the inclusion of children with disabilities in education, as this research highlights their low levels of participation. This finding has also been reported in previous studies [1], [4], [8], [14], [15], as well as a recent monitoring report of the Convention on the Rights of the Child [23]. The Millennium Development Goal of Universal Primary Education can only be achieved with this focus, and this is likely also to be the case for future Sustainable Development Goals on education. Furthermore, countries that are signatories of the UN Convention on the Rights of the Child or the UN Convention on the Rights of Persons with Disabilities cannot fulfil their responsibilities without inclusion of children with disabilities in education, as well as the necessity of addressing their right to health care.

A twin-track approach is widely advocated for promoting inclusion of children with disabilities – whether with respect to education, health care, or in other areas. This approach involves a focus on improving inclusion of children with disabilities in mainstream services as well as making specialist services available when needed. However, the evidence base on what works is currently very poor and needs to be strengthened substantially in order to identify scalable interventions [24], [25], - a recent review found only six intervention studies for children with disabilities in low and middle income countries [25]. Careful thought needs to be put into development and provision of interventions as these often requires engagement with many sectors. In addition, there are often family level impacts of childhood disability, which need to be considered when developing interventions [25].

With respect to inclusion in education, activities to promote inclusion may focus on strengthening the capacity of the education system to meet the needs of children with disabilities, as well as providing specialist services or support (e.g. Braille reading) for children with particular needs. Qualitative studies have identified strategies that seem to be effective in improving participation of children with disabilities in education [26], but more evidence is needed [24], [25]. Research is also needed to understand the barriers to uptake of education and the widespread exclusionary practices facing children with disabilities [26], [27] in order to identify strategies to overcome these barriers which may be setting specific. This study has identified a number of countries with very large disparities between children with and without disabilities that could provide useful information quite rapidly. Furthermore, there needs to be more research into understanding what the serious illnesses are which are more frequently experienced by children with disabilities to be able to better meet their health needs. Malnutrition showed a relationship with disability in some of the countries, which needs to be confirmed through an assessment of stunting and wasting among the sponsored children and may suggest the need for nutrition or feeding support programmes.

This study demonstrated that the Plan sponsorship dataset could be used for research purposes, which could be used to encourage other non-governmental organisations and agencies to use their available data for similar purposes.

In conclusion, children with disabilities in the Plan sponsorship programme are not fulfilling their educational potential, which is likely to have a long-term deleterious impact on their lives. These children also face the further challenge or greater vulnerability to serious illnesses. Mainstream development organizations need to focus on the inclusion of children with disabilities in order to meet their overall goals.

## Supporting Information

Table S1 **Distribution of type of impairment amongst Plan’s sponsored children reporting a disability.**
(DOCX)

Table S2 **Leading barriers to attendance at formal education, by disability status, amongst Plan’s sponsored children.**
(DOCX)

Table S3 **Association between poverty and disability, amongst Plan’s sponsored children.**
(DOCX)

## References

[1] World Health Organisation (2011) World Report on Disability. Geneva: World Health Organisation.

[2] UNICEF (2005) The State of the World’s Children 2006: Excluded and Invisible. New York: UNICEF.

[3] UNICEF (2013) State of the World’s Children 2013. New York: UNICEF.

[4] Filmer D (2008) Disability, Poverty, and Schooling in Developing Countries: Results from 14 household surveys. World Bank EconRev 22: 141–63.

[5] Singal N, Jeffery R (2011) Inclusive education in India: the struggle for quality in consonance with equity. In: Artiles A, Kozleski E, Waitoller F, editors. Inclusive Education: Examining Equity on Five Continents. Massachusetts: Harvard Education Press.

[6] Trani J-F, Browne J, Kett M, Bah O, Morlai T, et al (2011) Access to health care, reproductive health and disability: a large scale survey in Sierra Leone. Soc Sci Med 73: 1477–89. 22014873 10.1016/j.socscimed.2011.08.040

[7] Yousafzai AK, Filteau S, Wirz S (2003) Feeding difficulties in disabled children leads to malnutrition: experience in an Indian slum. Br J Nutr 90: 1097–106. 14641969 10.1079/bjn2003991

[8] Gottlieb CA, Maenner MJ, Cappa C, Durkin MS (2009) Child disability screening, nutrition, and early learning in 18 countries with low and middle incomes: data from the third round of UNICEF’s Multiple Indicator Cluster Survey (2005–06). Lancet 374: 1831–9. 19944864 10.1016/S0140-6736(09)61871-7

[9] Groce N, Kerac M, Farkas A, Schultink W, Bieler WB (2013) Inclusive nutrition for children and adults with disabilities. Lancet Glob Health 1: e180–e1. 25104341 10.1016/S2214-109X(13)70056-1

[10] United Nations (1989) Convention on the Rights of the Child. New York: United Nations.

[11] United Nations (2006) Convention on the rights of persons with disabilities. New York: United Nations.

[12] Bornstein MH, Hendricks C (2013) Screening for developmental disabilities in developing countries. Soc Sci Med97: 307–15. 10.1016/j.socscimed.2012.09.049 PMC3638080 23294875

[13] Filmer D, Pritchett LH (2001) Estimating wealth effects without expenditure data - or tears: an application to educational enrollment. Demography 38: 115–32. 11227840 10.1353/dem.2001.0003

[14] Rischewski D, Kuper H, Atijosan O, Simms V, Jofret-Bonet M, et al (2008) Poverty and musculoskeletal impairment in Rwanda. Trans R Soc Trop Med Hyg 102: 608–17. 18430444 10.1016/j.trstmh.2008.02.023

[15] Mont D, Nguyen C (2013) Does Parental Disability Matter to Child Education? Evidence from Vietnam. World Dev 48: 88–107.

[16] Alavi Y, Jumbe V, Hartley S, Smith S, Lamping D, et al (2012) Indignity, exclusion, pain and hunger: the impact of musculoskeletal impairments in the lives of children in Malawi. Disabil Rehabil 34: 1736–46. 22409227 10.3109/09638288.2012.662260

[17] World Bank (2008) Project appraisal document on a proposed credit to the People’s Republic of Bangladesh for a disability and children-at-risk project. Washington: World Bank.

[18] Wu L, Katz J, Mullany LC, Haytmanek E, Khatry SK, et al (2010) Association between nutritional status and positive childhood disability screening using the ten questions plus tool in Sarlahi, Nepal. J Health Popul Nutr 28: 585–94. 21261204 10.3329/jhpn.v28i6.6607 PMC2995027

[19] Nesbitt RC, Mackey S, Kuper H, Muhit M, Murthy GVS (2012) Predictors of referral uptake in children with disabilities in Bangladesh–exploring barriers as a first step to improving referral provision. Disabil Rehabil 34: 1089–95. 22136387 10.3109/09638288.2011.634943

[20] Nanoo N (2011) The Lives of Children with Disabilities in Africa: A Glimpse into a Hidden World. Addis Ababa: The African Child Policy Forum.

[21] Simkiss DE, Blackburn CM, Mukoro FO, Read JM, Spencer NJ (2011) Childhood disability and socio-economic circumstances in low and middle income countries: systematic review. BMC Pediatr 11: 119. 22188700 10.1186/1471-2431-11-119 PMC3259053

[22] United Nations, Population Division (2011) Sex Differentials in Childhood Mortality. New York: United Nations.

[23] United Nations (2011) Report of the Secretary- General on the Status of the Conventionon the Rights of the Child. New York: United Nations.

[24] Tomlinson M, Swartz L (2009) Officer A, Chan KY, Rudan I, et al (2009) Research priorities for health of people with disabilities: an expert opinion exercise. Lancet 374(9704): 1857–62. 19944866 10.1016/S0140-6736(09)61910-3

[25] Yousafzai AK, Lynch P, Gladstone M (2014) Moving beyond prevalence studies: screening and interventions for children with disabilities in low-income and middle-income countries. Arch Dis Child 0: 1–9. 10.1136/archdischild-2012-302066 24647995

[26] Singal N (2008) Working towards inclusion: Reflections from the classroom. Teach Teach Educ 24: 1516–29.

[27] Kalyanpur M, Gowramma I (2007) Cultural barriers to South Indian Families’ Access to Services and Educational Goals for Their Chlidren with Disabilities. J Int Ass Spec Educ 8: 69–82.

