# Supplementary material for: The Impact of Disability on the Lives of Children; Cross-Sectional Data Including 8,900 Children with Disabilities and 898,834 Children without Disabilities across 30 Countries
Source: PLoS One. 2014 Sep 9;9(9):e107300. doi: 10.1371/journal.pone.0107300 (PMC4159292; doi:10.1371/journal.pone.0107300)
Supplement: Table S1 — Distribution of type of impairment amongst Plan’s sponsored children reporting a disability. (DOCX) [file pone.0107300.s001.docx]

Web table 1: Distribution of type of impairment amongst Plan’s sponsored children reporting a disability

| Country | Learning | Physical | Communication | Vision | Hearing |
| --- | --- | --- | --- | --- | --- |
| South America |  |  |  |  |  |
| Bolivia | 75 (20%) | 71 (19%) | 121 (33%) | 75 (20%) | 30 (8%) |
| Brazil | 39 (27%) | 61 (42%) | 18 (13%) | 18 (13%) | 8 (6%) |
| Colombia | 68 (29%) | 50 (21%) | 60 (26%) | 44 (19%) | 13 (6%) |
| Dominican Republic | 29 (16%) | 57 (32%) | 35 (20%) | 49 (28%) | 8 (4%) |
| Ecuador | 39 (10%) | 144 (36%) | 124 (31%) | 68 (17%) | 22 (6%) |
| El Salvador | 62 (10%) | 232 (36%) | 215 (33%) | 95 (15%) | 42 (7%) |
| Guatemala | 48 (11%) | 105 (24%) | 132 (31%) | 118 (27%) | 29 (7%) |
| Honduras | 142 (26%) | 115 (21%) | 103 (19%) | 155 (28%) | 36 (7%) |
| Nicaragua | 95 (21%) | 102 (22%) | 129 (28%) | 105 (23%) | 29 (6%) |
| Paraguay | 23 (20%) | 32 (28%) | 26 (23%) | 28 (25%) | 5 (4%) |
| Peru | 50 (26%) | 42 (22%) | 47 (24%) | 48 (25%) | 8 (4%) |
|  |  |  |  |  |  |
| Africa |  |  |  |  |  |
| Benin | 1 (1%) | 34 (31%) | 5 (5%) | 50 (46%) | 18 (17%) |
| Egypt | 116 (26%) | 140 (31%) | 118 (26%) | 59 (13%) | 19 (4%) |
| Guinea | 5 (3%) | 63 (43%) | 47 (32%) | 19 (13%) | 12 (8%) |
| Kenya | 15 (6%) | 65 (25%) | 71 (28%) | 38 (15%) | 69 (27%) |
| Mozambique | 2 (2%) | 27 (23%) | 36 (30%) | 20 (17%) | 34 (29%) |
| Niger | 1 (1%) | 89 (48%) | 50 (27%) | 26 (14%) | 19 (10%) |
| Rwanda | 6 (3%) | 97 (45%) | 20 (9%) | 47 (22%) | 44 (21%) |
| Senegal | 10 (6%) | 42 (27%) | 69 (45%) | 27 (17%) | 7 (5%) |
| Sudan | 8 (6%) | 50 (38%) | 32 (24%) | 28 (21%) | 13 (10%) |
| Tanzania | 3 (3%) | 60 (57%) | 19 (18%) | 11 (10%) | 12 (11%) |
| Uganda | 4 (1%) | 97 (36%) | 69 (26%) | 40 (15%) | 58 (22%) |
| Zambia | 7 (6%) | 36 (32%) | 25 (22%) | 23 (20%) | 22 (19%) |
| Zimbabwe | 21 (11%) | 73 (37%) | 45 (23%) | 30 (15%) | 31 (16%) |
|  |  |  |  |  |  |
| Asia |  |  |  |  |  |
| India | 20 (4%) | 234 (45%) | 125 (24%) | 119 (23%) | 24 (5%) |
| Indonesia | 34 (9%) | 121 (32%) | 148 (39%) | 45 (12%) | 28 (7%) |
| Nepal | 8 (3%) | 136 (53%) | 64 (25%) | 41 (16%) | 10 (4%) |
| Philippines | 39 (10%) | 144 (36%) | 124 (31%) | 68 (17%) | 22 (6%) |
| Sri Lanka | 16 (10%) | 40 (24%) | 54 (33%) | 36 (22%) | 20 (12%) |
| Vietnam | 119 (20%) | 201 (33%) | 110 (18%) | 134 (22%) | 44 (7%) |
