# Supplementary material for: The Impact of Disability on the Lives of Children; Cross-Sectional Data Including 8,900 Children with Disabilities and 898,834 Children without Disabilities across 30 Countries
Source: PLoS One. 2014 Sep 9;9(9):e107300. doi: 10.1371/journal.pone.0107300 (PMC4159292; doi:10.1371/journal.pone.0107300)
Supplement: Table S2 — Leading barriers to attendance at formal education, by disability status, amongst Plan’s sponsored children. (DOCX) [file pone.0107300.s002.docx]

Web table 2: Leading barriers to attendance at formal education, by disability status, amongst Plan’s sponsored children

| Country |  | Children with disabilities | Children without disabilities |
| --- | --- | --- | --- |
| South America |  |  |  |
| Bolivia | Has an impairment | 57% | 0% |
|  | Too young | 20% | 79% |
| Brazil | Has an impairment | 50% | 0% |
|  | Too young | 46% | 97% |
| Colombia | Has an impairment | 50% | 1% |
|  | Too young | 8% | 79% |
| Dominican Rep | Has an impairment | 17% | 0% |
|  | Too young | 24% | 98% |
| Ecuador | Has an impairment | 73% | 0% |
|  | Too young | 10% | 62% |
| El Salvador | Has an impairment | 63% | 0% |
|  | Too young | 23% | 76% |
| Guatemala | Has an impairment | 61% | 0% |
|  | Too young | 20% | 58% |
| Honduras | Has an impairment | 56% | 0% |
|  | Too young | 17% | 54% |
| Nicaragua | Has an impairment | 82% | 0% |
|  | Too young | 8% | 58% |
| Paraguay | Has an impairment | 68% | 0% |
|  | Too young | 25% | 91% |
| Peru | Has an impairment | 56% | 0% |
|  | Too young | 13% | 84% |
|  |  |  |  |
| Africa |  |  |  |
| Benin | Has an impairment | 9% | 0% |
|  | Too young | 4% | 13% |
| Egypt | Has an impairment | 80% | 0% |
|  | Too young | 12% | 75% |
| Guinea | Has an impairment | 63% | 0% |
|  | Too young | 7% | 46% |
| Kenya | Has an impairment | 78% | 0% |
|  | Too young | 16% | 97% |
| Mozambique | Has an impairment | 30% | 0% |
|  | Too young | 63% | 95% |
| Niger | Has an impairment | 41% | 0% |
|  | Too young | 19% | 65% |
| Rwanda | Has an impairment | 21% | 0% |
|  | Too young | 74% | 97% |
| Senegal | Has an impairment | 42% | 0% |
|  | Too young | 13% | 53% |
| Sudan | Has an impairment | 69% | 0% |
|  | Too young | 10% | 90% |
| Tanzania | Has an impairment | 31% | 0% |
|  | Too young | 31% | 84% |
| Uganda | Has an impairment | 62% | 0% |
|  | Too young | 29% | 94% |
| Zambia | Has an impairment | 61% | 0% |
|  | Too young | 9% | 64% |
| Zimbabwe | Has an impairment | 61% | 0% |
|  | Too young | 32% | 72% |
|  |  |  |  |
| Asia |  |  |  |
| India | Has an impairment | 73% | 0% |
|  | Too young | 10% | 60% |
| Indonesia | Has an impairment | 73% | 0% |
|  | Too young | 15% | 79% |
| Nepal | Has an impairment | 73% | 0% |
|  | Too young | 21% | 61% |
| Philippines | Has an impairment | 74% | 0% |
|  | Too young | 14% | 63% |
| Sri Lanka | Has an impairment | 31% | 0% |
|  | Too young | 22% | 88% |
| Vietnam | Has an impairment | 81% | 0% |
|  | Too young | 8% | 68% |
