# Supplementary material for: The Impact of Disability on the Lives of Children; Cross-Sectional Data Including 8,900 Children with Disabilities and 898,834 Children without Disabilities across 30 Countries
Source: PLoS One. 2014 Sep 9;9(9):e107300. doi: 10.1371/journal.pone.0107300 (PMC4159292; doi:10.1371/journal.pone.0107300)
Supplement: Table S3 — Association between poverty and disability, amongst Plan’s sponsored children. (DOCX) [file pone.0107300.s003.docx]

Web table 3: Association between poverty and disability, amongst Plan’s sponsored children

|  | Poverty score | Children with disabilities | Children without disabilities | Age and sex adjusted OR^1^ (95% CI^2^) |
| --- | --- | --- | --- | --- |
| South America |  |  |  |  |
| Bolivia | 1 (poorest) | 98 (26%) | 10400 (25%) | Baseline |
|  | 2 | 94 (25%) | 10417 (25%) | 0·9 (0·7 - 1·2) |
|  | 3 | 86 (23%) | 10415 (25%) | 0·9 (0·6 - 1·1) |
|  | 4 (least poor) | 94 (25%) | 10386 (25%) | 0·9 (0·7 - 1·2) |
| Brazil | 1 (poorest) | 37 (26%) | 3218 (25%) | Baseline |
|  | 2 | 33 (23%) | 3216 (25%) | 0·8 (0·5 - 1·3) |
|  | 3 | 36 (25%) | 3213 (25%) | 0·9 (0·5 - 1·4) |
|  | 4 (least poor) | 38 (26%) | 3203 (25%) | 0·9 (0·6 - 1·4) |
| Colombia | 1 (poorest) | 60 (26%) | 5445 (25%) | Baseline |
|  | 2 | 53 (23%) | 5453 (25%) | 0·8 (0·6 - 1·2) |
|  | 3 | 63 (27%) | 5443 (25%) | 1·0 (0·7 - 1·4) |
|  | 4 (least poor) | 59 (25%) | 5444 (25%) | 0·9 (0·6 - 1·3) |
| Dominican Republic | 1 (poorest) | 45 (25%) | 7286 (28%) | Baseline |
|  | 2 | 48 (27%) | 5901 (22%) | 1·3 (0·9 - 2·0) |
|  | 3 | 42 (24%) | 6600 (25%) | 1·0 (0·7 - 1·6) |
|  | 4 (least poor) | 43 (24%) | 6595 (25%) | 1·0 (0·6 - 1·5) |
| Ecuador | 1 (poorest) | 124 (31%) | 8281 (25%) | Baseline |
|  | 2 | 93 (23%) | 8274 (25%) | 0·7 (0·6 - 0·9) |
|  | 3 | 100 (25%) | 8289 (25%) | 0·7 (0·6 - 1·0) |
|  | 4 (least poor) | 80 (20%) | 8302 (25%) | 0·6 (0·4 - 0·8) |
| El Salvador | 1 (poorest) | 154 (24%) | 8551 (25%) | Baseline |
|  | 2 | 159 (25%) | 8568 (25%) | 1·0 (0·8 - 1·3) |
|  | 3 | 178 (28%) | 8502 (25%) | 1·1 (0·9 - 1·4) |
|  | 4 (least poor) | 155 (24%) | 8547 (25%) | 1·0 (0·8 - 1·2) |
| Guatemala | 1 (poorest) | 82 (19%) | 9756 (25%) | Baseline |
|  | 2 | 111 (26%) | 9510 (25%) | 1·4 (1·1 - 1·9) |
|  | 3 | 127 (29%) | 9513 (25%) | 1·6 (1·2 - 2·1) |
|  | 4 (least poor) | 112 (26%) | 9586 (25%) | 1·3 (1·0 - 1·8) |
| Honduras | 1 (poorest) | 147 (27%) | 8363 (25%) | Baseline |
|  | 2 | 137 (25%) | 8373 (25%) | 0·9 (0·7 - 1·1) |
|  | 3 | 150 (27%) | 9228 (28%) | 0·9 (0·7 - 1·1) |
|  | 4 (least poor) | 117 (21%) | 7525 (22%) | 0·8 (0·6 - 1·0) |
| Nicaragua | 1 (poorest) | 114 (25%) | 6871 (25%) | Baseline |
|  | 2 | 106 (23%) | 6858 (25%) | 0·9 (0·7 - 1·1) |
|  | 3 | 137 (30%) | 6762 (25%) | 1·1 (0·9 - 1·4) |
|  | 4 (least poor) | 103 (22%) | 6846 (25%) | 0·8 (0·6 - 1·0) |
| Paraguay | 1 (poorest) | 18 (16%) | 2098 (27%) | Baseline |
|  | 2 | 34 (30%) | 1761 (23%) | 2·4 (1·3 - 4·2) |
|  | 3 | 28 (25%) | 1922 (25%) | 1·8 (1·0 - 3·2) |
|  | 4 (least poor) | 34 (30%) | 1918 (25%) | 2·1 (1·2 - 3·8) |
| Peru | 1 (poorest) | 42 (22%) | 6444 (26%) | Baseline |
|  | 2 | 28 (14%) | 6180 (25%) | 0·7 (0·4 - 1·1) |
|  | 3 | 58 (30%) | 6276 (25%) | 1·4 (0·9 - 2·0) |
|  | 4 (least poor) | 67 (34%) | 6272 (25%) | 1·6 (1·1 - 2·3) |
| Africa |  |  |  |  |
| Benin | 1 (poorest) | 37 (34%) | 6104 (25%) | Baseline |
|  | 2 | 29 (27%) | 6333 (26%) | 0·8 (0·5 - 1·2) |
|  | 3 | 28 (26%) | 5913 (24%) | 0·8 (0·5 - 1·3) |
|  | 4 (least poor) | 14 (13%) | 6095 (25%) | 0·4 (0·2 - 0·7) |
| Egypt | 1 (poorest) | 103 (23%) | 8365 (25%) | Baseline |
|  | 2 | 102 (23%) | 9237 (28%) | 0·9 (0·7 - 1·2) |
|  | 3 | 101 (22%) | 7694 (23%) | 1·1 (0·8 - 1·4) |
|  | 4 (least poor) | 146 (32%) | 8123 (24%) | 1·4 (1·1 - 1·9) |
| Guinea | 1 (poorest) | 57 (39%) | 8209 (29%) | Baseline |
|  | 2 | 31 (21%) | 6375 (23%) | 0·7 (0·4 - 1·1) |
|  | 3 | 37 (25%) | 8601 (31%) | 0·6 (0·4 - 0·9) |
|  | 4 (least poor) | 21 (14%) | 4877 (17%) | 0·6 (0·4 - 1·0) |
| Kenya | 1 (poorest) | 57 (22%) | 15201 (25%) | Baseline |
|  | 2 | 76 (29%) | 15731 (26%) | 1·3 (0·9 - 1·8) |
|  | 3 | 69 (27%) | 15715 (26%) | 1·2 (0·8 - 1·7) |
|  | 4 (least poor) | 56 (22%) | 13235 (22%) | 1·1 (0·8 - 1·6) |
| Mozambique | 1 (poorest) | 31 (26%) | 1850 (28%) | Baseline |
|  | 2 | 24 (20%) | 1579 (24%) | 0·9 (0·5 - 1·6) |
|  | 3 | 38 (32%) | 1579 (24%) | 1·4 (0·9 - 2·3) |
|  | 4 (least poor) | 26 (22%) | 1655 (25%) | 0·9 (0·5 - 1·6) |
| Niger | 1 (poorest) | 59 (32%) | 4822 (25%) | Baseline |
|  | 2 | 48 (26%) | 4856 (26%) | 0·8 (0·6 - 1·2) |
|  | 3 | 41 (22%) | 4520 (24%) | 0·7 (0·5 - 1·1) |
|  | 4 (least poor) | 37 (20%) | 4721 (25%) | 0·7 (0·4 - 1·0) |
| Rwanda | 1 (poorest) | 79 (37%) | 1931 (31%) | Baseline |
|  | 2 | 55 (26%) | 1227 (20%) | 1·1 (0·8 - 1·6) |
|  | 3 | 33 (15%) | 1520 (24%) | 0·5 (0·4 - 0·8) |
|  | 4 (least poor) | 47 (22%) | 1551 (25%) | 0·7 (0·5 - 1·1) |
| Senegal | 1 (poorest) | 43 (28%) | 8158 (25%) | Baseline |
|  | 2 | 35 (23%) | 8212 (25%) | 0·8 (0·5 - 1·2) |
|  | 3 | 36 (23%) | 8077 (25%) | 0·9 (0·5 - 1·3) |
|  | 4 (least poor) | 41 (26%) | 8141 (25%) | 1·0 (0·6 - 1·5) |
| Sudan | 1 (poorest) | 33 (25%) | 7113 (26%) | Baseline |
|  | 2 | 37 (28%) | 6991 (26%) | 1·3 (0·8 - 2·1) |
|  | 3 | 31 (24%) | 6223 (23%) | 1·2 (0·7 - 1·9) |
|  | 4 (least poor) | 30 (23%) | 6769 (25%) | 1·0 (0·6 - 1·7) |
| Tanzania | 1 (poorest) | 21 (20%) | 6067 (25%) | Baseline |
|  | 2 | 39 (37%) | 6047 (25%) | 1·8 (1·1 - 3·1) |
|  | 3 | 26 (25%) | 6094 (25%) | 1·2 (0·7 - 2·2) |
|  | 4 (least poor) | 19 (18%) | 5999 (25%) | 0·9 (0·5 - 1·8) |
| Uganda | 1 (poorest) | 87 (32%) | 9243 (26%) | Baseline |
|  | 2 | 72 (27%) | 8405 (24%) | 0·9 (0·6 - 1·2) |
|  | 3 | 66 (25%) | 10020 (28%) | 0·7 (0·5 - 0·9) |
|  | 4 (least poor) | 43 (16%) | 7530 (21%) | 0·6 (0·4 - 0·8) |
| Zambia | 1 (poorest) | 35 (31%) | 4351 (26%) | Baseline |
|  | 2 | 24 (21%) | 3969 (24%) | 0·7 (0·4 - 1·2) |
|  | 3 | 26 (23%) | 4207 (25%) | 0·7 (0·4 - 1·2) |
|  | 4 (least poor) | 28 (25%) | 4095 (25%) | 0·8 (0·5 - 1·3) |
| Zimbabwe | 1 (poorest) | 65 (33%) | 12126 (37%) | Baseline |
|  | 2 | 28 (14%) | 4456 (13%) | 1·2 (0·7 - 1·8) |
|  | 3 | 58 (29%) | 9456 (29%) | 1·1 (0·8 - 1·6) |
|  | 4 (least poor) | 49 (25%) | 7111 (21%) | 1·3 (0·9 - 1·8) |
| Asia |  |  |  |  |
| India | 1 (poorest) | 171 (33%) | 16191 (25%) | Baseline |
|  | 2 | 113 (22%) | 16268 (25%) | 0·6 (0·5 - 0·8) |
|  | 3 | 138 (26%) | 16139 (25%) | 0·7 (0·5 - 0·8) |
|  | 4 (least poor) | 100 (19%) | 16240 (25%) | 0·5 (0·4 - 0·7) |
| Indonesia | 1 (poorest) | 70 (19%) | 11426 (25%) | Baseline |
|  | 2 | 121 (32%) | 11434 (25%) | 1·7 (1·3 - 2·3) |
|  | 3 | 96 (26%) | 11252 (25%) | 1·4 (1·0 - 1·9) |
|  | 4 (least poor) | 89 (24%) | 11374 (25%) | 1·2 (0·9 - 1·7) |
| Nepal | 1 (poorest) | 68 (26%) | 9553 (25%) | Baseline |
|  | 2 | 80 (31%) | 9622 (25%) | 1·2 (0·9 - 1·6) |
|  | 3 | 52 (20%) | 9466 (25%) | 0·8 (0·6 - 1·1) |
|  | 4 (least poor) | 59 (23%) | 9552 (25%) | 0·9 (0·6 - 1·3) |
| Philippines | 1 (poorest) | 124 (31%) | 8281 (25%) | Baseline |
|  | 2 | 93 (23%) | 8274 (25%) | 0·7 (0·6 - 0·9) |
|  | 3 | 100 (25%) | 8289 (25%) | 0·7 (0·6 - 1·0) |
|  | 4 (least poor) | 80 (20%) | 8302 (25%) | 0·6 (0·4 - 0·8) |
| Sri Lanka | 1 (poorest) | 46 (28%) | 5390 (25%) | Baseline |
|  | 2 | 43 (26%) | 5393 (25%) | 0·9 (0·6 - 1·4) |
|  | 3 | 37 (22%) | 5436 (25%) | 0·8 (0·5 - 1·2) |
|  | 4 (least poor) | 40 (24%) | 5358 (25%) | 0·8 (0·5 - 1·2) |
| Vietnam | 1 (poorest) | 108 (18%) | 8581 (25%) | Baseline |
|  | 2 | 136 (22%) | 8508 (25%) | 1·2 (1·0 - 1·6) |
|  | 3 | 233 (38%) | 10600 (31%) | 1·5 (1·2 - 1·9) |
|  | 4 (least poor) | 131 (22%) | 6342 (19%) | 1·3 (1·0 - 1·7) |

^1^OR: odds ratio

^2^CI: Confidence interval
